# Community participation and consultation in palliative and end-of-life care: Building death literacy through a participatory theory of change

**DOI:** 10.1177/26323524261459462

**Published:** 2026-06-05

**Authors:** Yakubu Salifu, John Davies, Katie Eccles, Kofi Adu Gyamfi, Glenys Caswell

**Affiliations:** 1 International Observatory on End of Life Care, Division of Health Research, Faculty of Health and Medicine, 4396 University of Lancaster, Lancaster, United Kingdom; 2 COMPASS-Ghana (Compassionate Palliative Services) Charity, Bradford on Avon, United Kingdom; 3 Palliative Care Hub, Asamang SDA Hospital, Agona-Asamang, Ashanti Region, Ghana/ COMPASS-Ghana NGO, Agona-Ashanti, Ashanti Region, Ghana; 4 Independent Social Researcher and Death Studies Scholar, Nottingham, United Kingdom

**Keywords:** palliative care, health literacy, community participation, primary health care, public health, social support, Universal Health Coverage, health policy, Ghana

## Abstract

**Introduction:**

Death literacy refers to the knowledge, skills, confidence, and social capacity that enable individuals and communities to understand and respond to dying, death, and loss, including palliative and end-of-life care. In low- and middle-income countries such as Ghana, access to palliative care remains limited due to under-resourced health systems, poverty, and socio-cultural norms that discourage open discussions about death. Despite growing global interest in death literacy and public health palliative care, there is limited evidence on how these are developed through culturally embedded, community-led approaches in non-Western contexts.

**Objectives:**

To explore how the COMPASS-Ghana participatory intervention shaped community stakeholders’ understanding of death, dying, and palliative care, and how stakeholder engagement contributed to culturally relevant and sustainable service development.

**Design:**

Qualitative participatory study informed by a Theory of Change framework.

**Methods:**

Data were generated through three interlinked participatory intervention activities: a stakeholder engagement session involving 37 participants from healthcare, policy, faith-based, philanthropic, and community leadership backgrounds; a Theory of Change workshop with 25 participants; and focus group with 12 nursing and midwifery students. Data from facilitated discussions and participatory mapping were analysed using reflexive thematic analysis.

**Findings:**

Three interrelated themes were generated: (1) building shared language and awareness around death, (2) embedding palliative care within existing community structures, and (3) enhancing collective agency for sustainable care. The intervention supported a shift from viewing death as taboo to recognising it as a shared community concern, increased confidence in discussing end-of-life issues, and highlighted locally grounded strategies to address financial, caregiving, and service access barriers.

**Conclusion:**

Death literacy in Ghana emerges as a relational and collective process shaped through dialogue, trusted networks, and community action. Embedding palliative care within community and primary care structures aligns with public health approaches and supports Universal Health Coverage, responding to the World Health Assembly Resolution (WHA67.19).

## Plain language summary

In many parts of the world, including Ghana, people do not have access to the care they need at the end of life. Poor transport, limited health services, and cultural discomfort about talking openly about death can prevent individuals and families from preparing for the end of life. As a result, people often seek help very late, rely heavily on informal or traditional care, and experience unnecessary distress. The COMPASS-Ghana project aimed to address these challenges by working directly with communities, health workers, and local leaders to encourage open conversations about death and dying and to strengthen palliative care. Through community meetings, workshops, and discussions with nurses, doctors, faith leaders, and families, people were supported to understand what palliative care is and how it can help individuals and families. This community led approach helped people feel more confident talking about death, planning ahead, and supporting one another when someone is seriously ill. It also highlighted the importance of palliative care services that are culturally appropriate, affordable, and rooted in community life. The study found that building knowledge and confidence around death and dying—referred to as death literacy—through the COMPASS-Ghana project, involving communities in the design and delivery of care can lead to services that are more meaningful, sustainable, and aligned with people’s values.

## Introduction

Palliative and end-of-life care are increasingly recognised as essential components of global health systems, yet access remains highly unequal, particularly in low- and middle-income countries. In many resource-constrained settings, including sub-Saharan Africa, limited-service provision, workforce shortages, and socio-cultural factors restrict timely access to care.^1–3^ In addition, cultural taboos surrounding death and dying often inhibit open dialogue, advance care planning, and community engagement with palliative care.^4,5^ These challenges highlight the need for approaches that extend beyond clinical service delivery to include community participation and public understanding.

The concept of death literacy has emerged as a useful framework for addressing these gaps. Rooted in a public health approach to palliative care, death literacy refers to the knowledge, skills, and social capacity that enable individuals and communities to understand and respond to issues of dying, death, loss, and care.^6,7^ It emphasises that end-of-life care is not solely the responsibility of health professionals but is shared across families, communities, and wider society.^4^ However, most conceptualisations and empirical applications of death literacy have been developed in high-income, Western contexts, raising important questions about their relevance and transferability to non-Western settings.^7,8^

While existing literature demonstrates the importance of death literacy in improving communication, preparedness, and access to care,^6,8^ there remains limited empirical evidence on how death literacy can be developed in culturally diverse and resource-constrained contexts, particularly through participatory and community-led approaches. This lack of context-specific evidence represents a critical gap, as most interventions and conceptual frameworks are derived from health systems with greater resources and different socio-cultural dynamics.

In African contexts, where community networks, faith-based organisations, and family systems play a central role in caregiving,^9^ understanding how death literacy is constructed and enacted requires context-specific exploration. In this study, “community” refers to non-professional actors involved in care and support, including family members, faith leaders, local leaders, volunteers, and informal caregivers. In contrast, “healthcare professionals” refers to formally trained and regulated providers such as doctors, nurses, and allied health practitioners. Clarifying these distinctions is important in contexts where caregiving responsibilities are shared across formal and informal systems. While there is growing evidence on palliative care development in parts of Africa,^10,11^ there remains limited research examining how communities understand and engage with death, dying, and palliative care, particularly through participatory approaches. This gap limits the development of culturally grounded and sustainable models of care.

This paper addresses this gap by examining the COMPASS-Ghana model to palliative care development. The COMPASS-Ghana model is a community-embedded, participatory approach that integrates stakeholder engagement, Theory of Change processes, and local health system partnerships to co-develop culturally relevant palliative care solutions. This study explores how death literacy can be built within existing social and cultural structures by focusing on stakeholder engagement and locally driven processes. The study is guided by a conceptual framework that integrates death literacy,^7^ public health palliative care,^4,12^ participatory Theory of Change approaches,^13^ and a constructivist understanding of knowledge production, which recognises that knowledge about death and care is co-produced through social interaction, cultural practice, and lived experience. This framing directly informs the methodological choice of participatory engagement and qualitative inquiry. In doing so, it contributes to a broader understanding of how community participation can inform equitable and culturally responsive palliative care in resource-poor settings.

## Background

Recent developments in global palliative care have shifted attention from service availability alone to the broader social and structural conditions that shape engagement with end-of-life care. In low- and middle-income countries, particularly across sub-Saharan Africa, palliative care systems are evolving but remain unevenly integrated into national health frameworks, often concentrated in urban centres and reliant on limited specialist provision.^10,11^ As a result, care continues to be predominantly delivered within households and communities, where decision-making, caregiving, and bereavement support are embedded within social and cultural systems rather than formal healthcare structures. This has led to growing recognition of the need to understand how knowledge about death, dying, and care is generated and mobilised outside clinical settings.

Death literacy has gained traction as a framework for conceptualising this knowledge, with the Death Literacy Index, developed in Australia, now adapted and validated across diverse international settings, including the UK, Turkey, China, Sweden, Belgium, and the Netherlands.^6^ Empirical studies in Turkey and China further demonstrate its relevance in examining relationships between death literacy, uncertainty, death anxiety, and reflective practices among caregivers and healthcare professionals.^14–17^ While these developments strengthen the empirical foundation of the concept, they remain largely situated within high-income contexts with established public health approaches to palliative care.^18^ Moreover, existing applications tend to prioritise measurement and individual-level competencies, offering limited insight into how knowledge is collectively constructed and enacted within communities.

Emerging research increasingly emphasises that knowledge about death, dying, and care is relational, socially embedded, and co-produced within everyday interactions, rather than solely derived from formal health systems.^4,7,12^ In contexts where caregiving is primarily delivered through informal networks, such as families, faith communities, and local support systems, understandings of end-of-life care are shaped by cultural values, spiritual beliefs, and lived experience. Consequently, knowledge is dynamic and evolves through ongoing dialogue, shared practices, and collective meaning-making.^5,19^ Despite this, there remains a notable lack of empirical research exploring how these socially situated processes operate in African settings, where oral traditions, faith-based practices, and community authority structures play a central role in shaping perceptions and decision-making.

In response to these gaps, participatory approaches have increasingly been proposed as a means of generating contextually grounded knowledge while strengthening local capacity. Community engagement is a participatory research approach that involves stakeholders as active partners in shaping research processes, ensuring that interventions are contextually relevant, acceptable, and sustainable.^20^ Theory of Change is a structured participatory approach used to map how and why change is expected to occur, identifying assumptions, pathways, and outcomes collaboratively with stakeholders. Theory of Change methodologies, in particular, provide a structured yet flexible approach to mapping pathways of change, enabling stakeholders to articulate assumptions, identify barriers, and co-produce solutions.^13^ However, despite their growing application in global health, there is limited evidence on how such approaches contribute specifically to enhancing death literacy or transforming community engagement with palliative care.

The COMPASS-Ghana model represents a novel application of these principles, moving beyond awareness-raising towards a co-produced, community-embedded approach to knowledge development and service design.^21^ Rather than positioning communities as passive recipients of information, the model recognises stakeholders as active agents in shaping understanding, identifying priorities, and influencing care pathways, reflecting a broader shift towards recognising community systems as integral to sustainable palliative care development.

Against this backdrop, this study advances the field in three key ways. First, it moves beyond measurement-focused approaches to death literacy by examining how knowledge is socially constructed and negotiated within a specific cultural context. Second, it provides empirical insight into the role of participatory engagement in shaping community understandings of death, dying, and care. Third, it offers a contextually grounded account of how these processes can inform the development of culturally responsive and sustainable palliative care models in resource-constrained settings.

Therefore, this study is guided by the following research questions:

1. How do community stakeholders understand and interpret death, dying, and palliative care within the Ghanaian context?
2. How does participation in the COMPASS-Ghana model shape and transform these understandings?
3. How can participatory stakeholder engagement contribute to the co-production of culturally relevant and sustainable palliative care models?

## Aim

The aim of this study is to explore how the COMPASS-Ghana participatory intervention—comprising stakeholder engagement sessions, a Theory of Change workshop, and a student engagement session—shapes community understandings of death, dying, and palliative care, and to examine how these interlinked activities contribute to the co-production of culturally relevant and sustainable palliative care approaches.

## Methods

**Figure 1.**
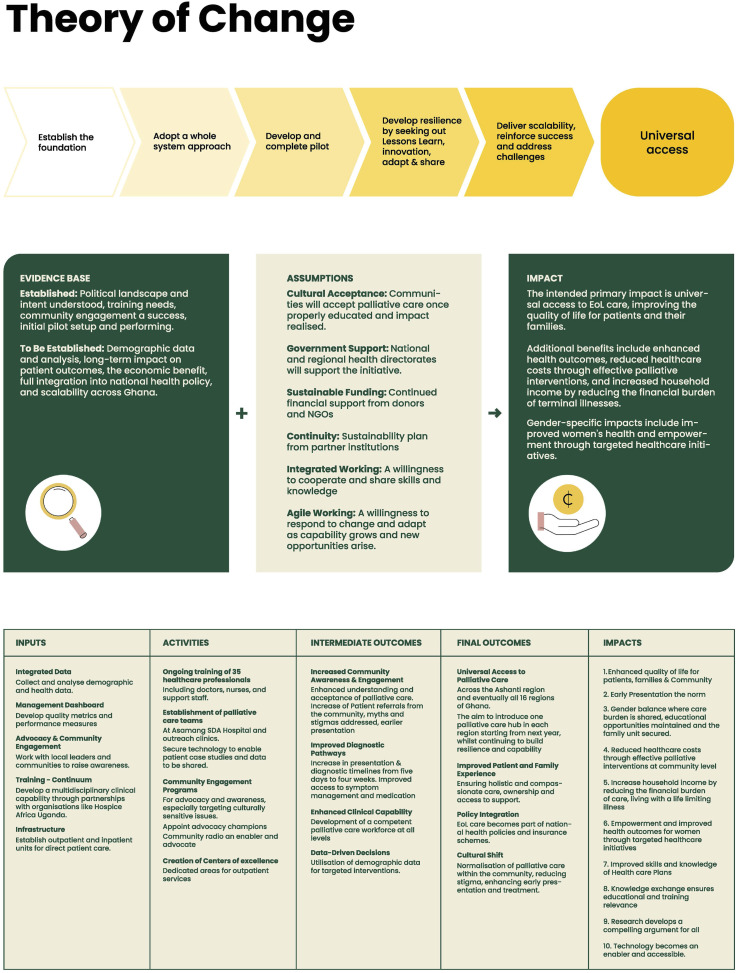
Theory of change.

This study adopted a qualitative approach to explore how the COMPASS-Ghana model influences community understandings of death, dying, and palliative care, and the role of participatory engagement in shaping service development. The study was underpinned by an interpretivist epistemology, recognising that meanings of death and care are socially constructed and contextually shaped.^22^ A participatory approach was employed, drawing on principles of co-production and Theory of Change to facilitate stakeholder engagement and collective knowledge generation (Figure 1).

### Study setting and context

This study was conducted in Ghana as part of the COMPASS-Ghana programme, a community-based initiative linked to Asamang Seventh-day Adventist (SDA) Hospital. The research was embedded within participatory community engagement activities, enabling exploration of real-world interactions between stakeholders, health systems, and socio-cultural contexts. The research team had an established collaborative relationship with local partners, which facilitated access, trust-building, and a nuanced understanding of the local context.

The study was primarily situated in the Ashanti Region at Asamang SDA Hospital, with activities conducted across both clinical and community-based settings. Additional stakeholder engagement activities were also undertaken in Accra (Greater Accra Region), allowing inclusion of national-level perspectives.

The target population comprised a diverse range of stakeholders with direct or indirect roles in shaping palliative and end-of-life care. This included community members, healthcare professionals, faith leaders, local policymakers, philanthropists, diaspora representatives, and patient and caregiver representatives. This multi-stakeholder setting reflects the resource-constrained and non-Westernised context of palliative care in Ghana, where access to formal services is limited and community networks play a central role in care provision.

### Inclusion criteria

(1) Adults aged 18 years and above;
(2) Individuals with professional, community, or lived experience relevant to death, dying, or palliative care;
(3) Willingness and ability to participate in stakeholder engagement activities.

### Exclusion criteria

(1) Individuals unable to provide informed consent;
(2) Individuals who are too unwell or otherwise unable to participate in engagement activities;
(3) Individuals for whom participation may pose undue distress or burden, as assessed by the research team or gatekeepers.

### Sample, sampling and sample size

Participants were purposively selected to ensure representation across stakeholder groups critical to the delivery, uptake, and development of palliative and end-of-life care. The sampling strategy prioritised diversity in gender, age, professional and community roles, and levels of prior engagement with palliative care, enabling the inclusion of a wide range of perspectives on death literacy and community participation. This approach is consistent with qualitative research methods that seek depth, variation, and contextual richness rather than representativeness.

The final sample comprised 74 participants across the three interlinked engagement activities. Given the qualitative and exploratory nature of the study, sample size was guided by established principles of qualitative research, where the aim is to achieve depth and richness of data rather than statistical generalisability.^22^ The inclusion of multiple stakeholder groups across different engagement settings enhanced the breadth and diversity of data collected.

Sample adequacy was determined through an iterative analytic process, whereby data were continuously reviewed across activities. Data sufficiency was considered achieved when no substantially new themes emerged from subsequent engagement sessions, indicating adequate coverage of the research questions and supporting the credibility of the findings.^23^

### Recruitment

Participants were recruited through established COMPASS-Ghana networks, including healthcare facilities, community organisations, and faith-based groups, to ensure inclusion of stakeholders relevant to the study aim of understanding and enhancing death literacy. Using purposive sampling, individuals with roles in caregiving, service provision, or community leadership were invited via email, telephone, and in-person contact. Information sheets were provided and explained verbally where needed. Written informed consent was obtained prior to participation. This approach ensured diverse perspectives on how knowledge, beliefs, and practices related to death and dying are formed and shared, enabling exploration of how the intervention influences death literacy across community and professional groups.

#### The intervention

In this qualitative study, ‘intervention’ refers to structured, theory-informed participatory activities designed to facilitate dialogue and influence understanding, rather than a clinical or experimental intervention. The participatory intervention comprised three interlinked engagement activities: (1) a Stakeholder Engagement Session, (2) a Theory of Change Workshop, and (3) a focus group with Nursing and Midwifery students, all informed by the COMPASS-Ghana Theory of Change model (Figure 1). In this study, intervention refers to designed, theory-informed activities to influence death literacy. The COMPASS-Ghana Theory of Change model and the topic guide (Table 1) provided the conceptual and practical framework guiding the design, sequencing, and integration of activities. The intervention adopted a consultative, community-led approach, combining stakeholder engagement with Theory of Change methodology.

**Table 1.** Semi-structured topic guide.

Topic area	Key prompts
Introduction	Purpose of discussion; voluntary participation; no right or wrong answers; participants may decline questions or withdraw.
Understanding death and dying	How death is discussed in the community; cultural or religious beliefs; comfort with death-related conversations.
Experiences of illness and care	Care received by people with serious illness; who provides care; challenges faced by families at end of life.
Awareness of palliative care	Prior understanding of palliative care; support needed at end of life; meanings of good end-of-life care.
Cultural and religious influences	Influence of beliefs on care decisions; important rituals or practices; ways beliefs support or challenge care.
Community roles and support	Roles of families, communities, and faith organisations; trusted sources of advice; opportunities for community involvement.
Barriers and challenges	Social, financial, and health system barriers; challenges in discussing or planning for death.
Improving care at end of life	Changes needed to improve care; how services can better support families; roles of professionals, communities, and policymakers.
Closing	Opportunity to share additional views on death, dying, care, or palliative care development.
Researcher notes	Guide used flexibly; probing questions used where appropriate; discussions facilitated sensitively and adapted to context.

The intervention was grounded in a consultative, community-led approach, combining stakeholder engagement with Theory of Change approach. Engaging communities through structured consultation was considered the most appropriate approach, as it ensured that the design, priorities, and strategies for improving death literacy were rooted in local knowledge, culturally relevant, and responsive to the lived experiences of patients, families, and community members.^24–26^

The three activities were designed to work sequentially and synergistically. The stakeholder engagement session elicited experiences, identified barriers, and initiated dialogue; the Theory of Change Workshop built on these insights to map pathways for sustainable palliative care development; and the student focus group provided additional perspectives to strengthen future workforce engagement. Together, these activities aimed to identify local priorities, co-develop strategies, and foster shared ownership of palliative care development.

A key feature of the stakeholder engagement session was the screening of a video produced by the Asamang SDA Palliative Care team. This visual narrative illustrated real-life patient and caregiver experiences, providing a culturally grounded entry point into discussions about death and dying. It helped to reduce stigma, break taboos, and stimulate open, reflective dialogue among participants.

### Data collection

Data were collected through three interlinked participatory activities: a stakeholder engagement session, a three-day Theory of Change workshop, and a student focus group. The stakeholder session facilitated discussions on access, affordability, and acceptability of palliative care, supported by a video illustrating patient and caregiver experiences. These participatory methods are appropriate as they enable collaborative knowledge production and are well established for generating rich, context-sensitive data in complex health research.^27–29^ The Theory of Change workshop enabled collaborative mapping of pathways for sustainable service development, generating data through group discussions and workshop artefacts. The student focus group session provided additional perspectives on integrating community-led palliative care into professional training.

All activities were guided by a semi-structured topic guide (Table 1) informed by existing literature on death literacy, the theory of change model, and public health palliative care. The guide was used flexibly, reviewed for cultural relevance, and refined iteratively during early engagement activities.

### Data analysis

All qualitative data were analysed using reflexive thematic analysis,^30^ following the COREQ reporting guidelines to ensure transparency and rigour.^31^ The COREQ checklist is provided in the Supplementary Material . Audio-recorded discussions were transcribed verbatim and, where necessary, translated from local languages (e.g., Twi) into English by bilingual researchers. Transcripts were anonymised and cross-checked to ensure accuracy and preserve meaning.^32,33^ Data from the three engagement activities were managed in NVivo and analysed iteratively using Braun and Clarke’s six-phase approach.^34^

The research team first familiarised themselves with the data through repeated reading of transcripts, field notes, and workshop artefacts. Line-by-line coding was then conducted inductively to capture meaningful features across datasets. In keeping with reflexive thematic analysis, coding was flexible and interpretive rather than applied within a fixed coding framework. Codes were compared, discussed, and refined through ongoing reflexive engagement within the research team to enhance depth of interpretation. Rather than constructing a rigid coding tree, codes were actively developed and shaped through the researchers’ analytic engagement with the data.

These codes were then grouped into candidate themes, which were reviewed against the full dataset to ensure coherence, relevance, and distinctiveness. Themes were subsequently defined, named, and interpreted in relation to death literacy and community-based palliative care, with attention to how meanings were co-constructed within the socio-cultural context of the study. Themes were developed inductively by the research team through iterative analysis and were not predefined a priori.

Analytic rigour was strengthened through an audit trail, reflexive team discussions, and triangulation across data sources. Emerging findings were also reviewed with participants and local collaborators (member checking) to enhance credibility and cultural sensitivity, while recognising that interpretations remained grounded in a reflexive rather than consensus-based analytic approach. Participants contributed beyond data provision through member-checking of emerging findings during engagement activities and by shaping priorities within the Theory of Change workshop, thereby influencing interpretation and co-production of recommendations.

### Rigour and reflexivity

The study adhered to established criteria for trustworthiness—credibility, dependability, confirmability, and transferability.^35^ Credibility was enhanced through triangulation of multiple data sources (stakeholder sessions, workshop outputs, and student engagement), member checking during engagement activities, and iterative discussions with local collaborators. Dependability and confirmability were supported through maintaining an audit trail of methodological and analytic decisions, while transferability was facilitated through detailed description of the study context.

Reflexivity was explicitly embedded throughout the research process, with careful consideration of the researchers’ dual roles, positionality, and potential influence on data generation and interpretation, particularly in sensitive end-of-life contexts.^36^ The research team (YS, KE, JD, KAG, and GC) brought multidisciplinary expertise in nursing, palliative care, sociology, and health leadership. All researchers had prior experience in qualitative research and/or palliative care practice, which informed both data collection and interpretation. The team critically examined their own positions and assumptions particularly in relation to working across cultural and professional contexts.

Reflexive bracketing was employed, whereby researchers actively reflected on and set aside preconceptions during data collection and analysis to remain open to participants’ perspectives. Regular reflexive meetings were held to discuss emerging interpretations and challenge assumptions, ensuring that findings remained grounded in the data. The researchers’ ongoing engagement with participants and local partners also supported transparency and accountability in interpretation, reducing the risk of imposing external perspectives on locally grounded experiences.

## Results

A total of 74 participants took part in the study, representing a diverse range of stakeholders involved in palliative and end-of-life care, including healthcare professionals, community leaders, policymakers, faith leaders, and students. This multi-sectoral composition reflects both formal and community-based perspectives. Detailed participant characteristics and roles are presented in Table 2. Across engagement activities, the themes reported below were evident in discussions involving participants from all major stakeholder groups, though perspectives varied in emphasis depending on participants’ roles, experiences, and social positions.

**Table 2.** Characteristics of participants.

Activity	No. of participants	Participant background (n)	Notes on contribution
Stakeholder Engagement Session	37	Nurses (5); Doctors (4); Allied health professionals (4); Member from Ghana Palliative Care Association (1), Faith leader/chaplain (1); Media (2), Corporate leaders/influencers (5); Patient & caregiver representatives (2); Philanthropists (3); Board of directors of COMPASS-Ghana NGO (4), Policymakers (2, including 1 Member of Parliament); International partners (4, including 1 UK FCDO representative).	Explored access, affordability, and acceptability of palliative care in line with the study aim, using the topic guide and Theory of Change framework; engaged with the Asamang SDA video to stimulate reflection on lived experiences; and identified collaborative partnerships to support community-informed development of sustainable, culturally relevant palliative care services.
Theory of Change Workshop	25	Nurses (6); Doctors (3); Hospital management (2); pharmacy technician, (2), Community leaders (2); other members of palliative care unit (4), COMPASS-Ghana programme lead (1); Chaplain/faith leaders (2), Nutrition officers (2), representative of Municipal Director (1)	Mapped stakeholder-informed inputs and resources to planned activities, short- and long-term outcomes, and intended impact within the COMPASS-Ghana Theory of Change model; identified data priorities to support monitoring and evaluation; and developed contextually relevant strategies for resource mobilisation and sustainability.
Nursing & Midwifery Student Session	12	Student nurses (8); Student midwives (4)	Shared perspectives on integrating palliative care into education and community engagement

### Themes

Analysis of data from the stakeholder engagement, Theory of Change workshop, and student consultations generated three overarching themes reflecting the influence of the COMPASS-Ghana model on community understanding of death, dying, and palliative care: (1) building shared language and awareness around death, (2) embedding palliative care within existing community structures, and (3) enhancing collective agency to support sustainable end-of-life care. These themes were evident across the majority of group discussions and participatory activities, with each theme being reiterated by multiple participants across different sessions, indicating shared experiences rather than isolated individual views.

#### Theme 1. Building shared language and awareness around death

Participants consistently reported that community conversations about death had been rare and often avoided due to cultural taboos and fear. This view was expressed by most community members and health professionals across stakeholder engagement sessions and workshops, particularly those with direct experience of end-of-life caregiving. The structured engagement sessions and video testimonies provided by the Asamang SDA Palliative Care team helped to normalise dialogue about dying and end-of-life needs. Many participants described the engagement process as transformative or “eye-opening”, noting that it enabled conversations about death that had previously felt socially prohibited. Community members, health professionals, and policymakers described the process as “eye-opening” and emphasised the value of using accessible, culturally resonant language to discuss care preferences and dignity at the end of life. The following quotes illustrate a shift in how death was discussed: These two quotes illustrate a transformative shift in how death is discussed and understood at the community level, which is central to the concept of death literacy.

“*Before this, we never spoke about death like this in public. Seeing our own people talk about it has made it easier for me to discuss it with my family*.” (Community Member, Stakeholder Engagement).

The COMPASS-Ghana model reframes death as a shared social concern rather than a private or taboo matter, thereby widening the circle of responsibility and support. This reframing was echoed by a substantial proportion of participants, particularly family caregivers and community representatives, who linked openness about death to reduced isolation and emotional burden. As one family member reflected:

*Before COMPASS-Ghana came we thought we had to carry this pain alone. Now we know we are not forgotten. Our mother died with dignity, and we were able to be by her side* (A family member).

These quotes demonstrate how culturally relevant and locally led dialogue can dismantle long-standing taboos around open discussion of dying. This is crucial because *death literacy* involves not only knowledge about end-of-life processes but also the confidence and social permission to engage in those conversations within one’s own cultural context.

*As doctors, we often avoid these conversations because we fear taking away hope, but this has shown me that honest conversations can actually build trust.* (Doctor)

*From a media perspective, we rarely tell stories about dying in a way that educates—this approach shows how powerful it can be to normalise these conversations publicly*. (Media representative)

While many participants expressed positive shifts in perspective others voiced discomfort about discussing death so openly. While the majority of participants reported increased openness and confidence in death-related discussions, a smaller number raised concerns about cultural appropriateness and emotional impact, particularly where families continued to hope for cure. These more cautious views sparked constructive dialogue and allowed the group to explore a middle ground between respecting cultural sensitivities and promoting openness about end-of-life care.

*The video showed us the reality without shame—it made me realise that planning for death is not a curse, but a way of protecting those we leave behind*. (Faith Leader, Stakeholder Engagement)

The faith leader in the above quote speaks to a reframing of death preparedness from a superstitious or negative act to one of care, responsibility, and love. This reflects a key dimension of *death literacy* as described by Noonan and colleagues^7^: the ability to interpret death-related information in a way that prompts practical and values-driven action. Here, the visual storytelling approach bypassed abstract fear and grounded the message in lived realities, empowering individuals to see planning as a proactive and protective measure.

*In pastoral care, we see how fear of death is deeply spiritual—these conversations help people find peace rather than fear.* (Chaplain/Faith leader)

*We need to learn how to talk about death in ways that respect belief but still prepare families—this is something we are not trained to do enough.* (Doctor)

Contrary, a few expressed discomforts. In contrast, a few participants expressed discomfort, reflecting persistent cultural beliefs: One community leader commented:

*In our culture, we believe that talking about death invites it, so it is not easy for us to discuss these things publicly* (community leader).

Another participant noted: *‘We fear that such conversations may upset families who are still hoping for a cure.’* These divergent perspectives were not dominant but were present across multiple sessions, prompting group discussions on balancing cultural sensitivity with openness. These concerns helped the group to explore ways of introducing death-related topics more sensitively, striking a balance between openness and cultural respect.

Together, these quotes underscore how the COMPASS-Ghana model leverages culturally resonant methods—community-led dialogue and locally produced media—to build the relational, emotional, and practical competencies needed for communities to engage more openly and constructively with death and dying. This progression is critical for reducing stigma, promoting early palliative care access, and fostering supportive environments for patients and families. Death literacy may include the knowledge to stop needless expensive and painful interventions including invasive scans. This contributes to patient’s false hopes rather the truth that the patient is dying, and a good holistic palliative care assessment is far more helpful.

#### Theme 2. Embedding palliative care within existing community structures

The COMPASS-Ghana model leveraged pre-existing social and faith-based networks to extend the reach and acceptability of palliative care services. This perspective was strongly shared among faith leaders, community influencers, and healthcare professionals, who consistently emphasised trust and familiarity as key facilitators of engagement. Stakeholders highlighted that religious leaders, former hospital staff, and local philanthropists played a crucial role in building trust and mobilising resources. Integrating palliative care into these networks was seen as a sustainable way to improve access in a context where formal services remain scarce. This reflects the *social action* of death literacy, as community members began identifying practical strategies to support patients and caregivers.

*We already have strong church networks here—if palliative care can work through them, then everyone will hear about it and feel safe to use it.* (Community Influencer, Stakeholder Engagement)

The role of faith organisations including churches cannot be understated. Many participants highlighted faith organisations as central channels for normalising palliative care, rather than viewing them as supplementary. The quote highlights the strategic role of trusted social institutions in building death literacy.

Religious networks in Ghana are not only sources of moral and spiritual guidance but also powerful channels for information dissemination and normalising conversations around death. And the media has a key role to play. The following quote illustrated how the religious group and the media helps in death literacy.

*Churches are not just places of worship—they are places where people seek guidance in crisis, so they are central to how we support families at the end of life.* (Chaplain/Faith leader)

*If we use platforms like radio and community media, we can reach people who may never come into the hospital.* (Media representative)

This aligns with evidence from African palliative care literature showing that leveraging faith-based structures can significantly reduce stigma and increase uptake of end-of-life services.

*Former staff abroad are ready to help; the challenge is connecting them directly to the work here, so they see the impact.* (Healthcare Professional, Theory of Change Workshop)

A participant admonished us to shifts attention to the transnational dimension of community engagement. While fewer participants spoke about diaspora involvement, those who did emphasised its strategic importance, particularly for sustainability and fundraising.

*As clinicians, we see the gaps every day—working with community leaders helps us bridge what the health system alone cannot provide* (allied health professional)

*Partnerships across sectors—health, media, community—are what make these services visible and trusted* (Media/communication stakeholder)

It underscores how diaspora networks can be mobilised for resource generation and advocacy, provided that communication and accountability channels are clear. Linking diaspora supporters directly to tangible local outcomes fosters sustained commitment, which is vital for long-term palliative care capacity in resource-poor settings. Capacity building occurred through stakeholder engagement, knowledge exchange, and skills development during participatory activities.

*Seeing the stories from our own communities made me realise that death is not just a family matter—it is a community responsibility.* (Stakeholder, Engagement session)

The above quote encapsulates a critical shift in collective mindset that underpins death literacy. This shift from private to collective responsibility was reported by most participants, indicating broad resonance across groups. Reframing death as a shared social concern rather than a private or taboo matter, the COMPASS-Ghana ‘*whole system approach’* to palliative and end of life care widens the circle of responsibility and support. This is essential for building Compassionate Communities in which end-of-life care is understood as a shared civic and moral responsibility rather than an isolated family burden.

These three quotes provide rich insights into how community structures, diaspora engagement, and collective responsibility intersect with death literacy in the COMPASS-Ghana model. Together, these perspectives demonstrate that death literacy in Ghana cannot be built solely through clinical services—it must be embedded in existing community fabrics, mobilise both local and diaspora networks, and shift cultural narratives about death toward collective care and responsibility.

#### Theme 3. Enhancing collective agency for sustainable care

The Theory of Change workshop revealed a strong sense of collective responsibility for sustaining palliative care services. This theme was particularly prominent among community leaders, healthcare professionals, and students, who focused on actionable steps rather than abstract ideals. Participants identified practical actions, such as hosting awareness events, implementing systematic data collection, and developing fundraising strategies. These suggestions emerged repeatedly across group work outputs, indicating shared priorities rather than isolated ideas.

The following illustrative quotes enforce the need for sustainable care.

*We must stop waiting for the government alone. This is our community, and we have to organise ourselves to care for our people at the end of life*. (Community Leader, Theory of Change Workshop)

The above quote demonstrates a transition from dependency on state systems to a proactive, community-led responsibility for end-of-life care. Although voluntary action is limited in many settings in Ghana, and when it exists it is usually ad hoc and reactive, participants suggested that paid community-based workers or ‘end-of-life navigators’ could be trained and supported to signpost services and act as trusted local sources of knowledge.

*I think as a people and me in leadership position, we need evidence not just for research’s sake, but to convince decision-makers that palliative care is a priority* (policy maker)

*If we tell these stories well and back them with data, we can mobilise both public support and funding. But that said, we need the communities to take action* (Media representative)

This aligns with death literacy principles that emphasise empowering local actors to take ownership of palliative care solutions, especially in environments where government infrastructure is limited or overstretched.

*If we can collect proper data about patients and caregivers, we can show the real need and use that evidence to get more funding* (Nursing Student, Asamang Engagement)

A trainee nurse in the above quote highlights the link between evidence generation and resource mobilisation. According to the student embedding data literacy within death literacy, communities strengthen their ability to advocate for themselves and attract sustainable funding, a point widely supported in public health literature on community-driven health systems strengthening in Africa.

*The workshop showed us that we already have many of the tools within our communities; we just need to connect them and act together*. (Healthcare Professional, Theory of Change Workshop)

The quote reinforces the concept that death literacy is not about importing entirely new systems but about recognising, valuing, and integrating existing assets.

*To me, I always hold the view that spiritual care must be part of the system—we cannot separate emotional, spiritual, and physical care at the end of life.* (Chaplain/Faith leader)

*Sustainability will come when communities, clinicians, and institutions all take shared responsibility rather than working in silos. (Corporate leader)*

This realisation fosters a sense of agency and collective capacity, which is critical for building resilient, sustainable palliative care networks. While this call reflects a strong sense of collective responsibility, participants also recognised that in contexts where formal volunteering is limited, community-based palliative care may need to be supported by paid or stipend community workers who can provide practical assistance, facilitate access to services, and act as local sources of end-of-life care knowledge.

### Integrative insight across themes

**Figure 2.**
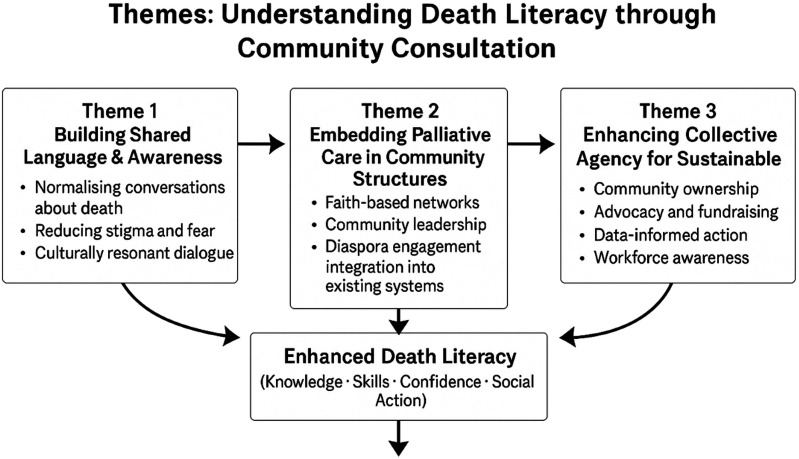
Interrelated themes demonstrating understanding of death literacy through community consultation.

Across all themes, findings demonstrate a progression from awareness to integration and action. Building shared language enabled open dialogue, which supported embedding palliative care within community structures and, in turn, strengthened collective agency. Together, these interconnected processes illustrate how death literacy is developed as a relational, collective, and contextually grounded practice. This integration is presented in Figure 2 below.

Using a public health palliative care and death literacy lens, the findings show how shared language builds social capital, enabling community-embedded palliative care and collective agency for sustainable service delivery.

## Discussion

This study set out to explore how participation in the COMPASS-Ghana model shaped community stakeholders’ understandings of death, dying, and palliative care, and how participatory engagement contributed to the co-production of culturally relevant and sustainable palliative care approaches. Importantly, the analytic approach was explicitly inductive, with themes emerging from stakeholder consultation data, workshop discussions, and participatory activities. These themes were subsequently interpreted through existing conceptual frameworks, including death literacy,^7^ public health palliative care,^5,12,37,38^ Compassionate Communities,^4^ and community-led palliative care in African contexts.^39–42^ This approach ensures that conclusions are grounded in empirical findings rather than imposed by theory, directly addressing concerns about unsupported claims or deductive overreach. COMPASS-Ghana model fostered open, culturally sensitive conversations about death and dying, contributing to shifts in understanding and engagement.

Three interrelated themes were identified: building shared language and awareness around death, embedding palliative care within existing community structures, and enhancing collective agency for sustainable care. Each theme corresponds to specific dimensions of death literacy—talking, knowing, doing, and acting socially—and provides insight into how death literacy is collectively produced in a resource-poor context. These themes are discussed below in direct relation to study findings and compared with existing literature.

### Building shared language and awareness around death

Findings from stakeholder engagement and community dialogue revealed that discussions about death had historically been avoided, shaped by cultural taboos, fear of misfortune, and spiritual interpretations. Participants described how structured engagement spaces, including facilitated discussions and visual narrative materials, enabled open but respectful conversations about dying and end-of-life care. These findings directly align with Theme 1, as the discussions suggested potential early shifts in participants’ confidence and openness around death, consistent with aspects of death literacy.^6,43^

Within the death literacy framework, this theme maps onto the domains of *knowledge* and *talking about death.*^7^ While the concept of a ‘good death’ was not directly elicited from participants, it is used here as an interpretive lens to situate findings within existing literature. Evidence from this study shows that when culturally resonant language and local narratives are used, individuals gain not only information but also social permission to speak about death within families and communities. Similar patterns have been observed in community conversation programmes in Uganda and Malawi, where dialogical approaches normalised death talk and facilitated earlier engagement with palliative services.^39,40,44–46^

The findings are further supported by research demonstrating that limited death literacy contributes to delayed help-seeking, minimal advance care planning, and heightened caregiver distress.^9,47,48^ Evidence from cross-cultural studies indicates that preparedness for death and engagement in anticipatory discussions can reduce caregiver death anxiety and support more positive bereavement outcomes. Similar patterns have been observed in Asian contexts, particularly in South Asia, where caregiving is often embedded within strong family structures and cultural norms that influence openness around end-of-life communication.^49–52^ Similarly, stronger family engagement and filial responsibility have been linked to lower caregiver burden.^53,54^

Importantly, this study contributes new insight by demonstrating that death literacy development in Ghana may begin not with technical information provision but with culturally grounded storytelling and facilitated dialogue. Rather than presenting death as a biomedical event, participants engaged with death as a social and relational experience—a finding that resonates with sociological work on “*good death*” as culturally situated rather than universal.^55–58^ The concept of good death in this study aligns with systematic review evidence that a “good death” is often shaped by relational and intergenerational caregiving contexts, where family presence, connection, and shared meaning are central.^59–61^ This study suggests that death literacy development in Ghana may emerge less from technical information provision and more from culturally grounded storytelling and facilitated dialogue, enabling communities to conceptualise a “good death” in locally meaningful ways, consistent with evidence that understandings of good end-of-life care are socially and culturally constructed.^62^ This matters for care because it shifts palliative care from a narrowly biomedical response to dying towards a relational, culturally situated practice centred on dignity, family presence, spiritual meaning, and community responsibility.

### Embedding palliative care within existing community structures

The second theme emerged strongly from participants’ emphasis on faith-based organisations, community leadership, diaspora networks, and informal social systems as critical to palliative care implementation. Stakeholders consistently identified churches, faith leaders, former healthcare workers, and community influencers as trusted intermediaries who could legitimise palliative care and reduce fear and mistrust. This theme reflects participants’ recognition that care must be embedded within pre-existing social infrastructures rather than delivered solely through formal health systems.

This finding aligns closely with public health palliative care principles, which emphasise using community development approaches to extend the reach of end-of-life care beyond clinical settings.^12,63,64^ African palliative care literature repeatedly highlights the importance of social trust and community networks in mediating access to services, particularly in rural and resource-constrained settings.^40,65–69^

In Ghana, where more than 70% of the population participates in faith-based activities,^70^ religious institutions function as central sites of social organisation, moral authority, and support. Embedding palliative care within these structures enhanced acceptability and reach, particularly among marginalised families who may distrust formal health services. Comparable findings have been reported in Malawi and Kenya, where faith-linked palliative interventions reduced stigma and facilitated early engagement.^42,71,72^ Studies highlight that even within clinical settings, discomfort, uncertainty, and limited preparedness around death persist among healthcare professionals, reinforcing the importance of strengthening death literacy and communication across both health systems and communities.^73,74^ Studies from both westernised and non-westernised context supports this finding by showing that effective community engagement in palliative care depends on intentionally building trust, clarifying roles, sharing information, and valuing non-professional contributors as legitimate members of the wider care network.^75,76^

The findings also resonate with evidence on home-based palliative care, which indicates that empowering families through knowledge and preparedness can improve outcomes while optimising limited resources.^65,77^ Interventions that support open discussion of personal experiences, involve trained facilitators, and engage families have been shown to reduce anxiety and depression among patients and caregivers.^78^

This study extends existing research by demonstrating that community structures do not merely facilitate service delivery but actively shape how palliative care is understood, legitimised, and enacted. Faith leaders and community influencers acted as cultural brokers, translating palliative care concepts into locally meaningful narratives. This addresses reviewer concerns by clearly linking the theme to both empirical findings and established literature.

The findings have important implications for universal health coverage (UHC), particularly in low-resource settings where access to specialist palliative care remains limited. By demonstrating how death literacy can be built through community engagement, trusted networks, and collective action, the COMPASS-Ghana model aligns with global calls to embed palliative care within primary health care and community systems as a core component of UHC. The World Health Assembly Resolution WHA67.19 explicitly calls on member states to integrate palliative care across health systems, including primary care and community levels, ensuring equitable access to quality end-of-life care as part of essential health services.^79,80^ The study findings show that when communities are equipped with shared language, cultural understanding, and practical capacity, palliative care becomes more accessible, acceptable, and affordable—key dimensions of UHC. Embedding palliative care within primary care and public health frameworks not only relieves pressure on overstretched hospitals but also strengthens continuity of care, family support, and cost-effective home-based services.^12,40,41^

In this way, death literacy functions as an enabling public health resource, supporting the realisation of UHC by linking community capability with formal health systems and reinforcing palliative care as an essential, not optional, component of comprehensive health coverage. This system-level integration also underpins sustainability and advocacy (Theme 3), as community-generated evidence and collective action can be mobilised to influence policy, secure funding, and sustain palliative care within UHC frameworks over time.

### Enhancing collective agency for sustainable care

The third theme reflects participants’ growing recognition of collective responsibility for sustaining palliative care services. Rather than positioning care as solely a governmental or professional obligation, community members described roles in advocacy, fundraising, data generation, and service coordination. This shift emerged most clearly during the Theory of Change workshop, where participants mapped pathways linking community action to sustainable outcomes.

This theme aligns strongly with the *social action* component of death literacy^7^ and with social capital theory, which conceptualises community capacity as emerging from networks, trust, and shared norms.^81^ Comparable findings have been reported in Zimbabwe and Kenya, where community-led initiatives sustained home-based care despite chronic underfunding.^82–84^

Gendered and generational dynamics also shaped collective agency. Recent qualitative research among African and Caribbean heritage families shows that cultural norms often discourage open discussion of dying, leaving adult children to make decisions without explicit guidance.^85^ Masculinity norms can further constrain emotional expression and caregiving engagement.^86–88^ Participants’ discussions in this study reflect awareness of these dynamics and the need for culturally sensitive approaches that support family dialogue and recognise diverse experiences of grief and care.^89,90^

The findings also resonate with Compassionate Communities’ literature, which emphasises strengthening informal care networks around dying individuals without replacing professional care.^4,12,64,91,92^ However, as noted by Dumont and colleagues,^93^ such models require careful contextual adaptation. This study demonstrates how Compassionate Community principles can be locally re-interpreted within Ghanaian socio-cultural realities rather than transferred wholesale from high-income contexts.

The Theory of Change process itself was instrumental in operationalising collective agency. Participants’ emphasis on data collection for advocacy aligns with broader African palliative care discourse highlighting the importance of locally generated evidence for influencing policy and funding.^40^ These findings suggest that strengthening death literacy within families and informal support networks including workplaces is critical for sustainable health systems, as these actors provide the primary emotional, practical, and social care that complements and extends limited formal services in resource-poor settings.^94,95^

## Implications for practice, policy, and research

The findings highlight the importance of developing palliative care in resource-constrained settings through community engagement, cultural relevance, and the strengthening of existing social networks. Integrating palliative care into primary healthcare and broader public health systems is essential to improve access, continuity, and equity of care. Health practitioners should build palliative care initiatives around trusted community structures to enhance reach and acceptability, while governments should formally recognise and incorporate community-led death literacy programmes within national palliative care strategies.

Further research work is needed to examine how participatory and community-driven models can be scaled and sustainably embedded within health systems. Longitudinal studies are particularly important to assess the impact of such approaches on access to care, quality of services, patient and family experiences, caregiver wellbeing, and cost-effectiveness.

### Strengths and limitations

A key strength of this study is the inclusion of diverse stakeholder perspectives, enabling a rich and contextually grounded understanding of palliative care in Ghana. The participatory design allowed for the co-production of knowledge, enhancing relevance and applicability. However, the study has limitations. The findings are context-specific and may not be directly transferable to other settings without consideration of cultural and health system differences. Additionally, data were generated through group-based engagement activities, which may have influenced the depth or openness of individual contributions.

## Conclusion

This study provides empirically grounded insight into how community-based participatory engagement can shape understandings of death, dying, and palliative care in a resource-constrained Ghanaian context. Through an explicitly inductive analytic approach, three interconnected themes were identified—building shared language and awareness, embedding palliative care within community structures, and enhancing collective agency—each reflecting core dimensions of death literacy.

Taken together, the findings suggest that death literacy in this setting is not simply an individual cognitive attribute but a relational and collective process, produced through dialogue, trusted networks, culturally shared meanings, and practical community action.

Positioning palliative care within familiar social structures and recognising the value of community knowledge, the COMPASS-Ghana model demonstrates how public health palliative care principles can be meaningfully adapted to local contexts. At the same time, these findings should not be over-generalised. The study was conducted within a specific Ghanaian region shaped by particular faith systems, leadership structures, and health-service relationships. Transferability therefore depends on careful cultural adaptation rather than direct replication. Nevertheless, the study offers a robust conceptual and empirical foundation for understanding how death literacy can be cultivated through participatory, community-embedded approaches in resource-poor settings. In summary, the COMPASS-Ghana model illustrates that sustainable palliative care development may be strengthened when communities are engaged not as passive recipients of services but as active partners in defining needs, shaping understanding, and mobilising collective responses to death and dying.

## Supplemental material

Supplemental material - Community participation and consultation in palliative and end-of-life care: Building death literacy through a participatory theory of change Supplemental material for Community participation and consultation in palliative and end-of-life care: Building death literacy through a participatory theory of change by Yakubu Salifu, John Davies, Katie Eccles, Kofi Adu Gyamfi, and Glenys Caswell in Palliative Care and Social Practice.

## Data Availability

The primary data that support the findings of this study are available from the corresponding author upon reasonable request. However, ENTREQ quality appraisal have been included as Supplemental Materials for transparency and auditability.*
