# Supplementary material for: Community participation and consultation in palliative and end-of-life care: Building death literacy through a participatory theory of change
Source: Palliat Care Soc Pract. 2026 Jun 5;20:26323524261459462. doi: 10.1177/26323524261459462 (PMC13241610; doi:10.1177/26323524261459462)
Supplement: Supplemental material - Community participation and consultation in palliative and end-of-life care: Building death literacy through a participatory theory of change [file sj-pdf-1-pcr-10.1177_26323524261459462.pdf]

**Consolidated criteria for reporting qualitative studies (COREQ): 32-item Checklist**

| No. | Item Guide questions/description | **Page no.** |
|---|---|---|
| **Domain 1: Research team and reflexivity** | | |
| *Personal Characteristics* | | |
| *1. Interviewer/facilitator* | **Which author/s conducted the interview or focus group?**<br><br>YS | Page 5 |
| *2. Credentials* | **What were the researcher's credentials? E.g., PhD, MD**<br><br>YS –*PhD, MPhil, BSc, RN, DipEd, DipN, SFHEA, RN*<br>KE – RN (specialist palliative care care nurse)<br>JD- MBA, BA<br>KAG- MBA, BA<br>GC- PhD, MA, BA | |
| *3. Occupation* | **What was their occupation at the time of the study?**<br><br>YS registered nurses and<br>Lecturer and CEO, COMPASS-Ghana<br>KE- Nurse, CNO COMPASS-Ghana<br>JD- COO COMPASS-Ghana<br>KAG- Hospital Administrator, and COMPASS-Ghana project manager.<br>GC- Sociologist, academic and Palliative care researcher in the UK | N/A |
| *4. Gender* | **Was the researcher male or female?**<br><br>YS, JD and KAG– males<br>KE and GC – females | N/A |
| 5. Experience and training | **What experience or training did the researcher have?**<br><br>YS-has a certificate in qualitative research methods from the University of Alberta-Canada.<br>KE, JD, and KAG- Have years of cinical experience<br>GC- Senior Research Fellow. They are both experienced qualitative researchers in palliative care with several publications | N/A |
| *Relationship with participants* | | |
| 6. Relationship established | **Was a relationship established prior to study Commencement?**<br><br>YS- Is a clinician-researcher was but did not have any direct prior contact with the participants.<br>KAG- Is the the COMPASS-Ghana and has | |

| | | |
|---|---|---|
| | relationship with the participants but was not involved in recruitment or interview.<br>KE and JD- had no relationship with participants.<br>GC is  also experienced, researcher who is non-clinician and brought in fresh, unbiased perspectives. | |
| 7. Participant knowledge of the interviewer | **What did the participants know about the researcher? e.g. personal goals, reasons for doing the research**<br><br>Participants did not know researchers but were told of the goal of the research. | Page 10 |
| 8. Interviewer characteristics | **What characteristics were reported about the interviewer/facilitator? e.g. Bias, assumptions, reasons and interests in the research topic**<br><br>These have been provided above. | |
| | | |
| **Domain 2: study design** | | |
| *Theoretical framework* | | |
| 9. Methodological orientation and Theory | **What methodological orientation was stated to underpin the study? e.g. grounded theory, discourse analysis, ethnography, phenomenology, content analysis**<br><br>This was a qualitative with interpretivist epistemology. | Page 7 |
| *Participant selection* | | |
| 10. Sampling | **Sampling**<br>**How participants were selected? e.g.**<br><br>Purposive sampling | Page |
| 11. Method of approach | Three enegagement sessions | Page 9-10 |
| 12. Sample size | 74 | Page 7 |
| 13. Non-participation | NA | |
| *Setting* | | |
| 14. The setting of data collection | **Where was the data collected? e.g. home, clinic, workplace**<br><br>School (student nurses), and clinic (theory of change workshop), and hired place (stakeholder's session) | Page 9-10 |
| 15. Presence of nonparticipants | **Was anyone else present besides the participants and researchers?**<br><br>N/A | |
| 16. Description of sample | **What are the important characteristics** | Pages 6-7 |

| | | |
|---|---|---|
| | **of the sample? e.g. demographic data, date**<br><br>These have been provided on the stated pages. | |
| *Data collection* | | |
| 17. Interview guide | **Were questions, prompts, guides provided by the authors? Was it pilot tested?**<br><br>Yes, semi-structured interview guides were developed by the authors based on the study aims and relevant literature. The guides were reviewed within the research team and pilot-tested with a small number of participants to ensure clarity and relevance, with minor refinements made prior to data collection. | See interview guide submitted as part of supplementary list |
| 18. Repeat interviews | **Were repeat interviews carried out? If yes, how many?**<br><br>No | N/A |
| 19. Audio/visual recording | Yes | Page 7 |
| 20. Fieldnotes | Yes | Page 8 |
| 21. Duration | Average of 1 hour student enegagement, and and 2-3 hours theory workshop and stakeholder enegagements. | Page 8 |
| 22. Data saturation | **Was data saturation discussed?**<br><br>Yes. Data saturation (or data sufficiency) was considered and achieved, as no new themes emerged from the data . | Page 8 |
| 23. Transcripts returned | **Were transcripts returned to participants for comment and/or correction?**<br><br>No. Transcripts were not returned to participants for comment or correction. Instead, participant engagement was incorporated through member checking of emerging findings, consistent with the study's interpretivist epistemological position and reflexive thematic analysis approach, which does not assume a single fixed "correct" transcript interpretation. | N/A |
| **Domain 3: analysis and findings** | | |
| *Data analysis* | | |
| 24. Number of data coders | **How many data coders coded the data?**<br><br>YS did all the coding and was reviewed by KE, JD and GC. | Pages 11 |
| 25. Description of the coding tree | **Did authors provide a description of the coding tree?** | Page 5 |

| | | |
|---|---|---|
| | Yes. A description of the coding framework (coding tree) has been provided in the manuscript, outlining the development of themes and sub-themes. | |
| 26. Derivation of themes | **Were themes identified in advance or derived from the data?**<br><br>Themes were derived inductively from the data through a reflexive thematic analysis process. | Page 18 |
| 27. Software | **What software, if applicable, was used to manage the data?**<br><br>Nvivo | Page 10 |
| 28. Participant checking | **Did participants provide feedback on the findings?**<br><br>No, this was not in line with author's philosophical underpinning | N/A |
| *Reporting* | | |
| 29. Quotations presented | **Were participant quotations presented to illustrate the themes/findings? Was each quotation identified? e.g. participant number**<br><br>Yes. Quotations were used to illustrate themes and were identified using participant pseudonyms, with representation across participants | Pages 12 to 18 |
| 30. Data and findings consistent | Yes | Pages 6-11 |
| 31. Clarity of major themes | Yes-three major themes | Page 6 |
| 32. Clarity of minor themes | N/A | Pages 18 |
